# Automated recognition of objects and types of forceps in surgical images using deep learning

**DOI:** 10.1038/s41598-021-01911-1

**Published:** 2021-11-19

**Authors:** Yoshiko Bamba, Shimpei Ogawa, Michio Itabashi, Shingo Kameoka, Takahiro Okamoto, Masakazu Yamamoto

**Affiliations:** 1 Department of Surgery, Institute of Gastroenterology, Tokyo Women’s Medical University, 8-1, Kawadacho Shinjuku-ku, Tokyo, 162-8666 Japan; 2 Ushiku Aiwa Hospital, Ibaraki, Japan; 3 Department of Surgery 2, Tokyo Women’s Medical University, Tokyo, Japan

**Keywords:** Cancer, Gastroenterology, Medical research, Mathematics and computing

## Abstract

Analysis of operative data with convolutional neural networks (CNNs) is expected to improve the knowledge and professional skills of surgeons. Identification of objects in videos recorded during surgery can be used for surgical skill assessment and surgical navigation. The objectives of this study were to recognize objects and types of forceps in surgical videos acquired during colorectal surgeries and evaluate detection accuracy. Images (n = 1818) were extracted from 11 surgical videos for model training, and another 500 images were extracted from 6 additional videos for validation. The following 5 types of forceps were selected for annotation: ultrasonic scalpel, grasping, clip, angled (Maryland and right-angled), and spatula. IBM Visual Insights software was used, which incorporates the most popular open-source deep-learning CNN frameworks. In total, 1039/1062 (97.8%) forceps were correctly identified among 500 test images. Calculated recall and precision values were as follows: grasping forceps, 98.1% and 98.0%; ultrasonic scalpel, 99.4% and 93.9%; clip forceps, 96.2% and 92.7%; angled forceps, 94.9% and 100%; and spatula forceps, 98.1% and 94.5%, respectively. Forceps recognition can be achieved with high accuracy using deep-learning models, providing the opportunity to evaluate how forceps are used in various operations.

## Introduction

Recently, artificial Intelligence (AI) has been extensively utilized in many fields^1^ and has contributed tremendously to improvements and advancements of technology. In this context, development using deep-learning technology^2,3^ has shared in the contribution. Deep learning is based on computer programs that automatically conduct repetitive learning from provided data and identify appropriate rules based on this process^4,5^. In the medical field, convolutional neural networks (CNNs)^6,7^ have also been extensively used in recent years not only for saving and archiving endoscopic surgical videos but also for analyzing the data from operations. The object recognition model used in this study has been commonly used to diagnose retinal diseases^8,9^, skin cancer^10–13^, colorectal neoplasms in endoscopy^14,15^, and arrhythmia in electrocardiography^16–18^. This research is expected to improve surgeons’ knowledge and professional skills^19^.

By analyzing preoperative images and intraoperative procedures and returning useful information to the surgeon during an operation, optimal surgery for patients that avoids risk through surgical navigation is the ultimate ideal. As a first step in the analysis of surgical procedures, an object recognition model is required to identify objects in surgical videos that require surgical skill assessment and surgical navigation. Attempts to develop such an object recognition model have been made, but sufficient results have not yet been obtained^8^. Herein, we constructed a model to recognize the object and types of forceps in surgical videos acquired during colorectal surgeries and evaluated its accuracy.

## Materials and methods

### Institutional approval

The protocol for this study was reviewed and approved by the Tokyo Women’s Medical University Review Board (Protocol No: 5380) and conducted according to the principles of the Declaration of Helsinki. All datasets were encrypted, and the identities of the patients were protected.

### Consent to participate

Oral consent was obtained from all study subjects. Informed consent forms that include information on the purpose of the study and study methods, the subject, the name of the implementing organization, the name of the person in charge, and how to handle personal information were obtained and captured in the electronic medical records. For all other research subjects, information will also be disclosed by posting a document approved by the Ethics Committee on the Tokyo Women's Medical University website; this posting will also mention the possibility to refuse to participate as a research subject.

### Datasets

The colorectal surgical videos used for annotation were recorded during surgeries conducted at the Tokyo Women’s Medical University. A total of 1173 images were extracted from 11 surgical videos for model training, and another 500 images were extracted from 6 additional videos for validation. The following 5 types of forceps in the videos were selected for annotation: grasping, ultrasonic, clip, angled (Maryland and right-angled), and spatula forceps. A surgical video with a 60 s run time was extracted from the other videos and used to verify the model.

### Analysis

The software IBM Visual Insights^20^ (Power SystemAC922; NVIDIA Tesla V100 GPU, 32 GB) was used for the CNN for deep learning. It includes the most popular open-source deep-learning framework and tools, and is built for easy and rapid deployment. The modeling types included in the software are GoogLeNet, Faster R-CNN, tiny YOLO V2, YOLO V3, Detectron, Single Shot Detector (SSD) and Structured segment network (SSN). Detecrton was selected for use in this study. IBM Visual Insights automatically splits the dataset for internal validation of the model’s performance during training. The default value of 80/20 will result in the use of 80% of the test data (at random) for training and the use of the remaining 20% for measurements/validation.

### Imaging data and model deployment

**Figure 1:**
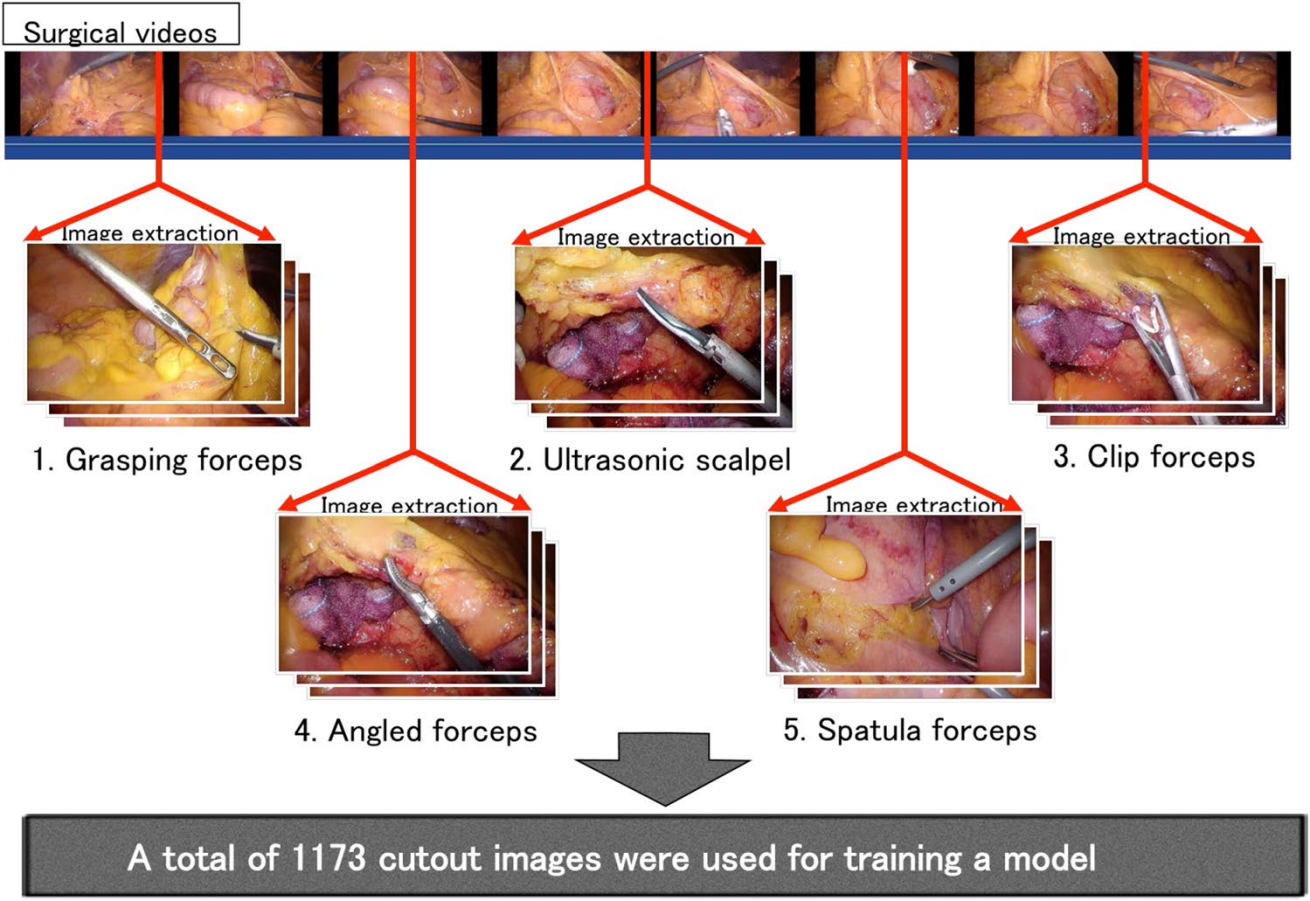
Extraction of still images from surgical videos for data labeling. Five types of forceps, namely, grasping forceps, ultrasonic scalpel clip forceps, angled forceps (Maryland and right angle), and spatula forceps, were annotated in these images.

**Figure 2:**
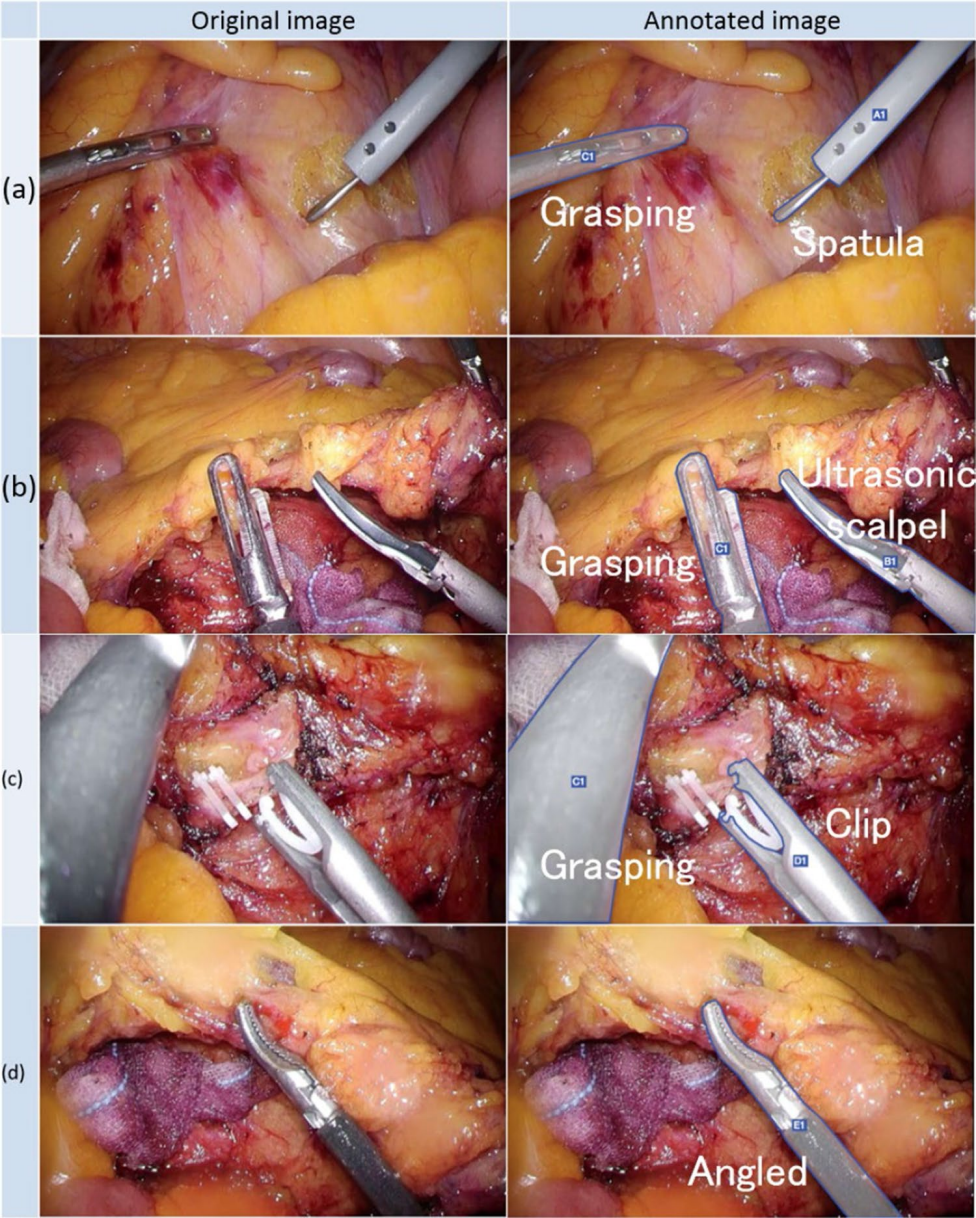
Representative images of labeled forceps. Five types of forceps, namely, grasping forceps, ultrasonic scalpel, clip forceps, angled forceps, and spatula forceps, were selected and labeled in the extracted images to create a forceps-type recognition model. The images on the left side are original, and the images on the right side show the annotated forceps.

**Figure 3:**
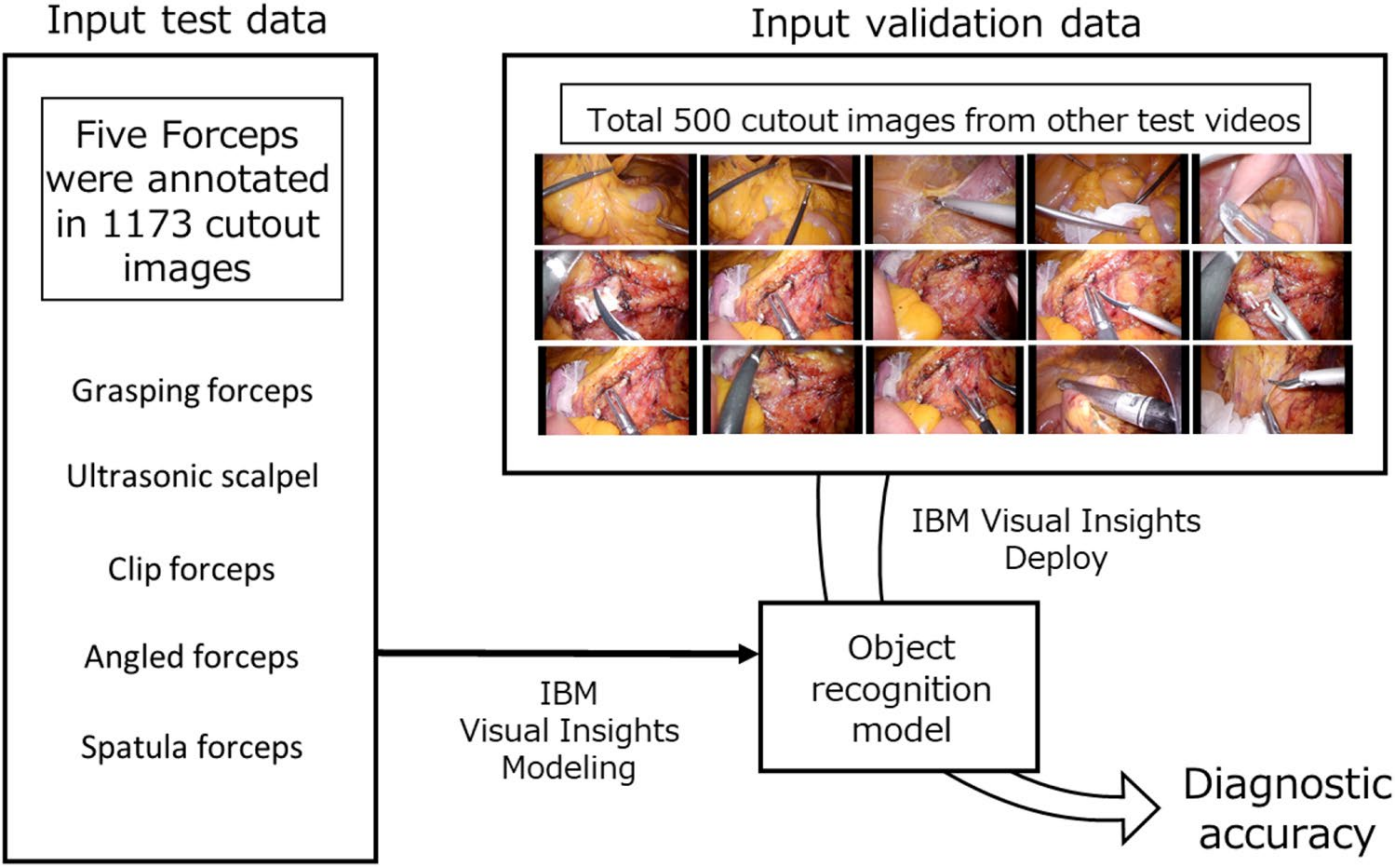
Flow of analysis using IBM Visual Insights. The five selected types of forceps were labeled in 1173 extracted images to create a forceps-type recognition model. Another 500 cutout images used for validation were input in the model to verify whether each type of forceps was recognized accurately.

Abdominal endoscopic images were extracted from surgical videos (Fig. 1). In total, 1173 images were extracted to train a forceps-type recognition model. Five types of forceps were selected for manual annotation by only 1 researcher. The selected types of forceps were grasping forceps, ultrasonic scalpel, clip forceps, angled forceps, and spatula forceps (Table 1 and Fig. 2). The model was deployed, and the other 500 test images of various different angles of forceps with different patterns were input into the deployed model to verify its diagnostic accuracy (Fig. 3).

**Table 1:** Number of annotated forceps.

Forceps	Number of annotated forceps
Grasping forceps	903
Ultrasonic scalpel	311
Clip forceps	138
Angled forceps	185
Spatula forceps	281
Total	1818

### Performance metrics

Accuracy: percentage of correct image labels.

Mean average precision (mAP): calculated mean of precision for each object.

Precision: percentage of images with a correctly labeled object out of all labeled images that contain an object.

Recall: percentage of images that are labeled to contain an object out of all tested images that contain an object.

Intersection over Union (IoU): location accuracy of the image label boxes.

Confidence score: event probability.

## Results

**Figure 4:**
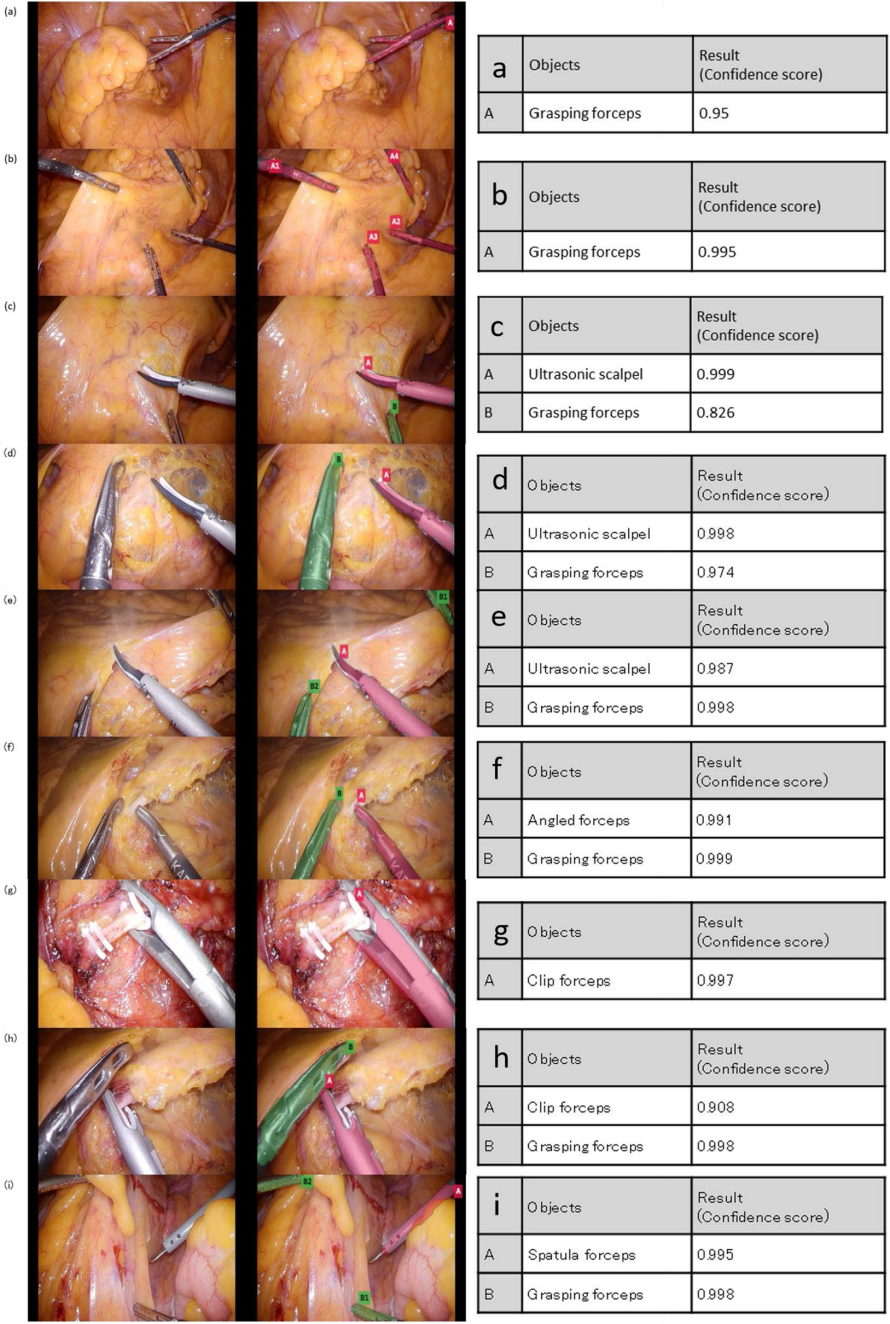
Representative images demonstrating accurate results. The images on the right side are original. The images in the middle are test results. The images on the right side show the confidence scores of each result.

The accuracy, mAP, precision, recall, and IoU of the model were 90%, 100%, 92%, 100%, and 77%, respectively (Fig. 4).

The total number of forceps identified in 500 test images was 1062. Of these, the number of correctly detected forceps was 1039 (97.8%). The number of false positives was 31. The recall and precision of each type of forceps calculated from the outcome values were as follows: grasping forceps, 98.1% and 98.0%; ultrasonic scalpel, 99.4% and 93.9%; clip forceps, 96.2% and 92.7%; angled forceps, 94.9% and 100%; and spatula forceps, 98.1% and 94.5%, respectively (Table 2).

**Table 2:** Test results for each type of forceps, and corresponding recall and precision.

Forceps	Number of forceps in images	Number of forceps identified correctly	Number of forceps not identified	False positive	Recall (95% CI)	Precision (95% CI)
Grasping	648	636	12	13	98.1% (97.1–99.2)	98.0% (96.9–99.1)
Ultrasonic scalpel	170	169	1	11	99.4% (98.3–100.6)	93.9% (90.4–97.4)
Clip	53	51	2	4	96.2% (91.1–101.4)	92.7% (85.9–99.6)
Angled	138	131	7	0	94.9% (91.3–98.6)	100% (100–100)
Spatula	53	52	1	3	98.1% (94.5–101.8)	94.5% (88.5–100.5)
Total	1062	1039	23	31	97.8% (97.0–98.7)	97.1% (96.1–98.1)

A surgical video with a 60 s run time was used to test the model, with the results indicating that the object was detected accurately (Supplementary Information).

## Discussion

In the field of surgery, AI-based decision support systems have provided a broad range of technological approaches to augment the information available to surgeons that have accelerated intraoperative pathology and surgical step recommendations^19^. Accurate and efficient object representation and segmentation are necessary for multilabel object classification in surgery based on the annotation of objects and frameworks^21^. Further, skill and motion assessments in surgical videos using CNN have been reported in recent years^22–24^.

In this study, we demonstrated the recognition of forceps (including type of forceps) from surgical images using CNN. In most test results, all 5 types of forceps were detected correctly with high confidence scores. Correspondingly, we obtained positive results in terms of the corresponding recall and precision values. The trained model was able to accurately detect the forceps at various angles (Fig. 4a–i). These results indicate that the model recognized the shapes and colors of each type of forceps with high precision.

**Figure 5:**
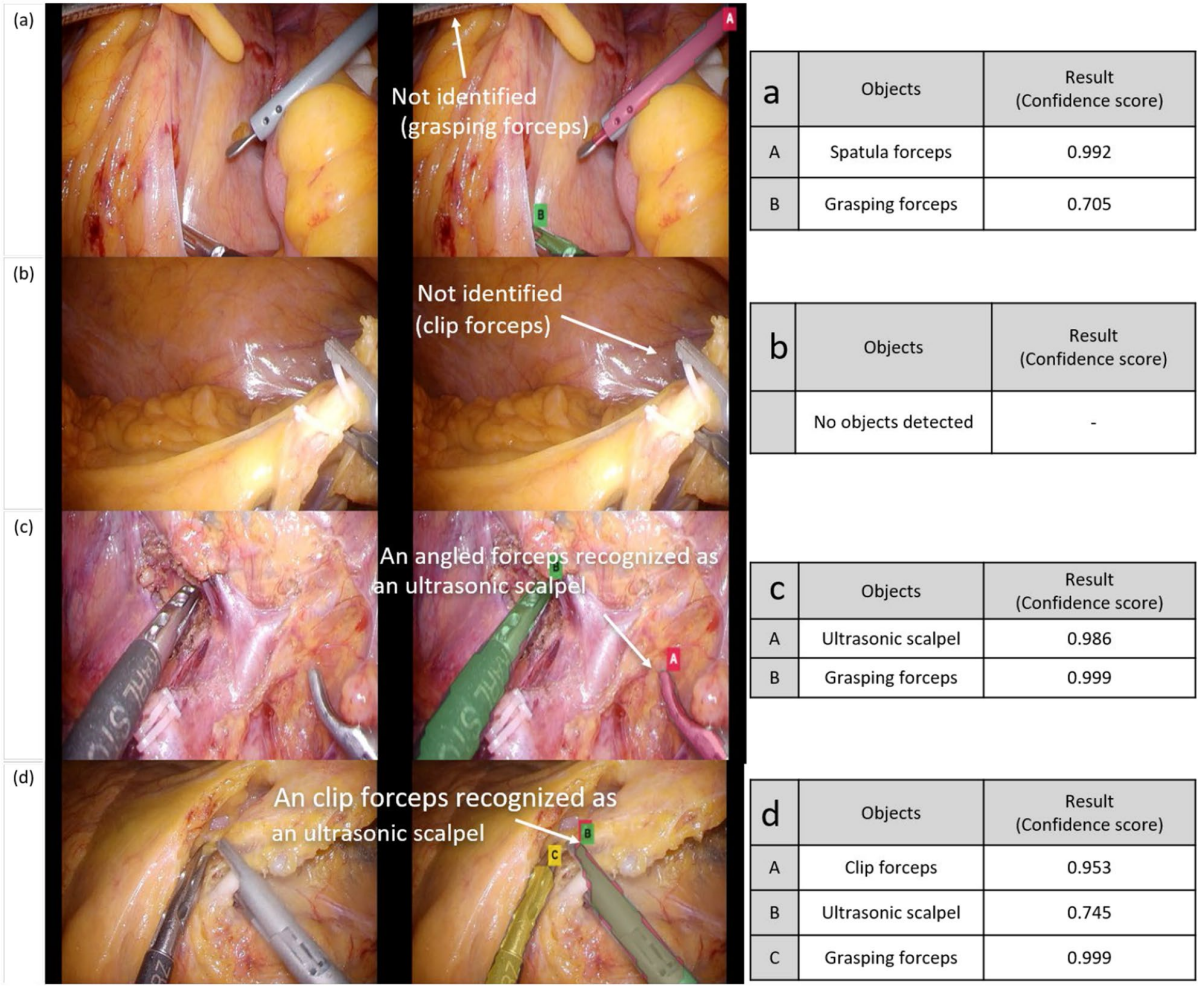
Representative images demonstrating inaccurate results. The images on the right side are original. The images in the middle are test results. The images on the right show the confidence scores of each result. (**a**) A grasping forceps and 1 spatula forceps were detected accurately, but 1 of the 2 grasping forceps in the image was not detected correctly; (**b**) the clip forceps was not identified correctly; (**c**) the angled forceps was recognized as an ultrasonic scalpel incorrectly; and (**d**) the clip forceps was identified correctly but was also recognized as an ultrasonic scalpel.

Although small in number, some forceps were not detected, or the outcomes yielded false positives. Based on the incorrect outcome images, we found that errors arose when only part of the forceps was observed in the images (Fig. 5a,b) or when the shapes of the forceps were similar to those of other types of forceps (Fig. 5c,d). Additionally, the results suggest that image resolution affects the validation outcome considerably. Because the forceps are in motion during surgeries, they are sometimes blurred in surgical videos or are closed in the cutout images. As a result, the model could not identify them or would recognize them as another type of forceps.

The potential of automatic video indexing and surgical skill assessment has been reported with the use of 300 laparoscopic sigmoidectomy videos from multiple institutions in Japan^25^. In the present study, the recall and precision values were good despite the limited learning because of the mixed frameworks of deep learning based on the use of the commercial software IBM Visual Insights.

The results of our study will aid the development of a system that will manage, deliver, and retrieve surgical instruments for surgeons upon request. The object recognition model in surgery has reached feasible performance levels for widespread clinical use. The object recognition of forceps could be used to provide real-time object information during surgeries upon further development based on the results of this study. By integrating and developing these technologies, the digitalization of surgical scenes and techniques becomes possible. The ability to evaluate how and what procedure was performed is significant. Moreover, these innovations will enable surgical technique evaluation and surgical navigation. Utilization of AI is largely expected not only in medical treatments, such as the prevention and diagnosis of diseases, but also in cases associated with insufficient resources and in risk management to prevent medical accidents.

This study had some limitations. First, it is difficult to modify the model itself via tuning other than by changing the training data, because the model was made using IBM Visual Insights. Further, there were only limited types of forceps created from colorectal cancer videos of a single facility.

## Conclusion

In this study, we evaluated the recognition of different types of forceps using CNN and obtained positive results with high accuracy. Results of this study demonstrate the opportunity to evaluate use and navigation of forceps in surgeries.

## Supplementary Information

Supplementary Video 1.
